# Transcriptional landscape of pathogen‐responsive lncRNAs in rice unveils the role of ALEX1 in jasmonate pathway and disease resistance

**DOI:** 10.1111/pbi.13234

**Published:** 2019-09-11

**Authors:** Yang Yu, Yan‐Fei Zhou, Yan‐Zhao Feng, Huang He, Jian‐Ping Lian, Yu‐Wei Yang, Meng‐Qi Lei, Yu‐Chan Zhang, Yue‐Qin Chen

**Affiliations:** ^1^ Guangdong Provincial Key Laboratory of Plant Resources State Key Laboratory for Biocontrol School of Life Sciences Sun Yat‐Sen University Guangzhou China

**Keywords:** long noncoding RNA, bacterial blight, jasmonate pathway, ALEX1, disease resistance

## Abstract

Plant defence is multilayered and is essential for surviving in a changing environment. The discovery of long noncoding RNAs (lncRNAs) has dramatically extended our understanding of post‐transcriptional gene regulation in diverse biological processes. However, the expression profile and function of lncRNAs in disease resistance are still largely unknown, especially in monocots. Here, we performed strand‐specific RNA sequencing of rice leaves infected by *Xanthomonas oryzae* pv. *Oryzae* (*Xoo*) in different time courses and systematically identified 567 disease‐responsive rice lncRNAs. Target analyses of these lncRNAs showed that jasmonate (JA) pathway was significantly enriched. To reveal the interaction between lncRNAs and JA‐related genes, we studied the coexpression of them and found 39 JA‐related protein‐coding genes to be interplayed with 73 lncRNAs, highlighting the potential modulation of lncRNAs in JA pathway. We subsequently identified an lncRNA, ALEX1, whose expression is highly induced by *Xoo* infection. A T‐DNA insertion line constructed using enhancer trap system showed a higher expression of ALEX1 and exerted a significant resistance to rice bacterial blight. Functional study revealed that JA signalling is activated and the endogenous content of JA and JA‐Ile is increased. Overexpressing ALEX1 in rice further confirmed the activation of JA pathway and resistance to bacterial blight. Our findings reveal the expression of pathogen‐responsive lncRNAs in rice and provide novel insights into the connection between lncRNAs and JA pathway in the regulation of plant disease resistance.

## Introduction

Plants possess a highly sophisticated and effective endogenous defence system to withstand pathogens and herbivores, referred to as plant innate immunity, which is fundamental for plants to survive and adapt to environmental challenges in nature. Advances in the las.t decade have broadened our understanding of transcriptome‐wide rewriting of plant genomes during invasion, signal perception, defence‐gene activation and expression of resistance responses (Buscaill and Rivas, 2014). Many of these responses are regulated by an array of cross‐communicating signal transduction pathways, within which noncoding RNAs (ncRNAs) fulfil important roles (Chaloner *et al*., 2016; Xu *et al*., 2018). An example is that flg22‐induced miR393 expression could negatively regulate its target genes, the F‐box auxin receptor *TIR1*,* AFB2* and *AFB3* mRNAs, which in turn contribute to the resistance of Arabidopsis to the bacterium *Pseudomonas syringae* by repressing auxin signalling (Navarro *et al*., 2006). NB‐LRR (nucleotide binding and leucine‐rich repeat) genes are major classes of plant innate immune receptors. Numerous miRNAs were found to regulate NB‐LRR gene expression in diverse plants. A subset of these miRNAs triggered phasi‐RNA synthesis from targeting *NB‐LRR* genes to potentiate their silencing effect (Deng *et al*., 2018a; Fei *et al*., 2013; Yu *et al*., 2018).

In addition to small noncoding RNAs, transcriptome studies in plants evidenced that genomes transcribe a multitude of long noncoding RNAs (lncRNAs), which are longer than 200 nt in length and do not have discernable coding potential (Chekanova, 2015). Recent progress has indicated that lncRNAs have functions associated with virtually every biological process in different organisms (Deng *et al*., 2018b; Ulitsky, 2016). Until now, only a few lncRNAs have been functionally characterized with regulatory mechanisms clearly stated in plants, including COLDAIR, COOLAIR and an antisense lncRNA MAS in the regulation of flowering (Castaings *et al*., 2014; Heo and Sung, 2011; Zhao *et al*., 2018), LDMAR in the regulation of anther development (Ding *et al*., 2012), ELENA1 in modulating plant immunity (Seo *et al*., 2017), LAIR expression in increased rice grain yield (Wang *et al*., 2018b) and IPS1 in phosphate metabolism (Shin *et al*., 2006). More intriguingly, accumulating evidence has also showed that lncRNAs are of great importance in the responses of plants against abiotic and biotic stresses (Liu *et al*., 2015). In Arabidopsis, the expression profiles of 1832 lncRNAs have been found to be significantly altered under drought, cold, high‐salt and/or abscisic acid (ABA) treatment (Liu *et al*., 2012a). *Fusarium oxysporum* infection in Arabidopsis triggers a number of lncRNAs changing their expression, and several of these lncRNAs are identified to play important roles in antifungal immunity (Zhu *et al*., 2014). In addition, a comprehensive disease‐responding lncRNA expression in defence against a cotton fungal disease was also reported (Zhang *et al*., 2018). These findings suggest that diverse sets of lncRNAs confer essential functions in the development and stress responses of a set of plant species.

*Xanthomonasoryzae* pv. *oryzae* (*Xoo*) is an aggressive rice pathogenic bacterium that causes serious bacterial leaf blight, the most devastating bacterial disease in rice worldwide. The interaction between rice and *Xoo* has already been widely studied, and knowledge of their relationship is considered a model system for studying plant host–bacterial pathogen interactions. Emerging data have revealed that protein‐coding genes play important roles in rice defence responses to *Xoo*, with potential prospects for crop breeding in order to control or eliminate the damage of bacterial blight to rice production (Ke *et al*., 2017; Liu *et al*., 2014; Zhang and Wang, 2013). Small noncoding RNAs involved in the regulation of rice responses to the invasion of *Xoo* had been identified on the genome scale, which revealed a large number of differentially expressed miRNAs and defence‐responsive mRNAs in rice‐*Xoo* interactions (Hong *et al*., 2015). Nevertheless, it is largely unknown whether lncRNAs are also involved in this biological process.

In this study, we infected the rice flag leaves with *Xoo* strain PXO99A, collected samples at 0, 2, 6, 12 and 24 h post‐infection (hpi) and performed strand‐specific whole transcriptome sequencing of rice samples. We systematically identified 567 rice lncRNAs, with a specific focus on the lncRNAs that were differentially expressed at different time points. The GO and KEGG pathway analyses of the putative lncRNA‐targeted genes definitely showed that the jasmonate (JA) pathway is significantly influenced during lncRNA‐mediated rice defence responses, and a regulatory network between lncRNAs and JA‐associated genes was also constructed. We further identified an lncRNA, ALEX1, which is specifically expressed in *Xoo*‐infected rice leaves. The rice mutant with elevated expression of ALEX1 exerts significant resistance to *Xoo* pathogen. Functional studies reveal that the up‐regulation of ALEX1 in mutant could activate JA signalling and increase endogenous JA accumulation. Overexpression of ALEX1 in rice also confirmed the activation of JA pathway and rice resistance to *Xoo* pathogen. Our findings reveal the involvement of lncRNAs in plant defence responses and provide further information to connect lncRNAs with the JA pathway in the regulation of plant innate immunity.

## Results

### Genome‐wide identification of rice lncRNAs responsed to *Xoo* infection

**Figure 1:**
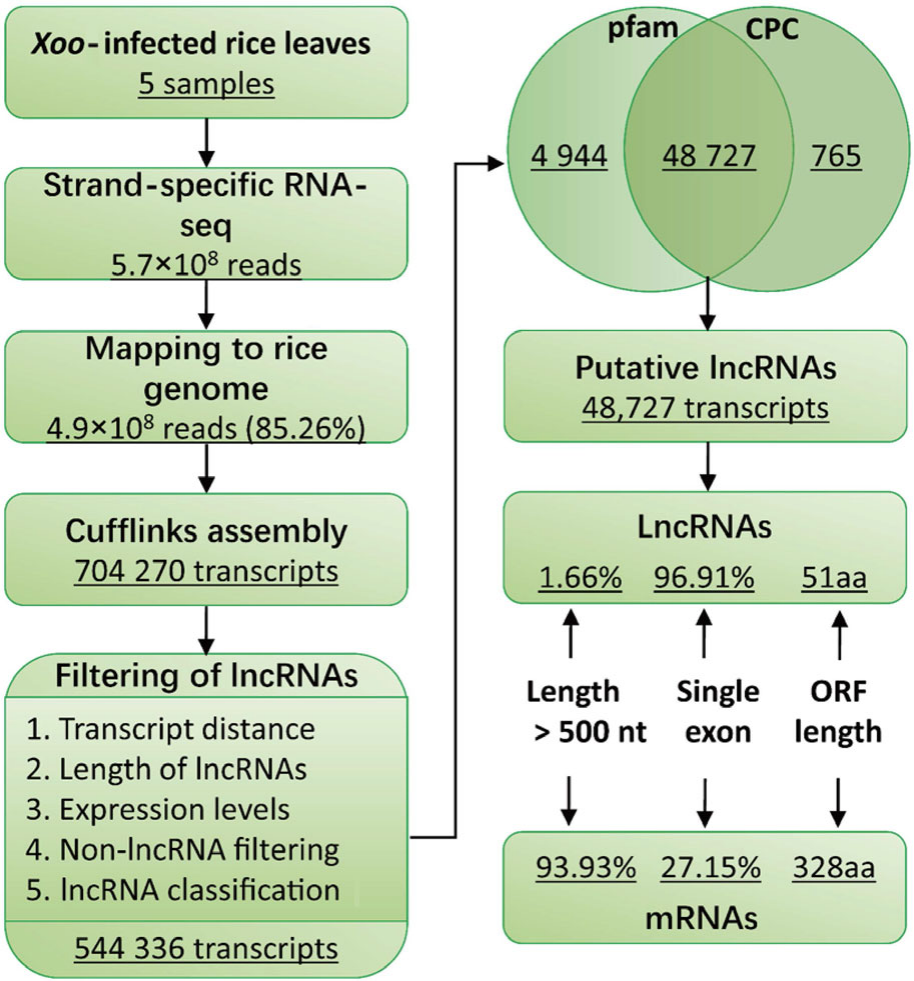
Pipeline of lncRNA identification in *Xoo*‐infected rice flag leaves and comparison of lncRNAs and mRNAs. Pfam, a database of protein families; CPC, coding potential calculator; ORF, open reading frame.

To characterize a comprehensive set of lncRNAs expressed during the infection of *Xoo* in rice, we first inoculated rice flag leaves with the *Xoo* strain PXO99A and collected the treated leaves at 0, 2, 6, 12 and 24 hpi. Strand‐specific paired‐end deep sequencing was performed, and 5.7 × 10^8^ reads were generated, of which 85.26% could be mapped to the referenced rice genome sequences (Figure 1).

The Cufflinks packages were used to assemble the transcriptomes, and 704 270 combined transcripts were expressed in at least one of the five sample data sets. Because lncRNAs are longer than 200 nt and have non‐protein‐coding potential, we set strict criteria to identify the lncRNA transcripts (see the Experimental procedures section) and finally obtained 48 727 transcripts that were the high‐confidence putative lncRNAs (Figure 1). To compare the putative lncRNAs identified in this study with mRNAs, we explored their genomic characteristics and expression profiles. Only 1.66% (807 of 48 727) putative lncRNAs were more than 500 nt in length, while 93.93% (39 777 of 42 346) mRNAs were longer than 500 nt; 96.91% (47 221 of 48 727) putative lncRNAs were single‐exon transcripts, while the percentage for mRNAs was 27.15% (11 497 of 42 346). We also compared the lengths of the open reading frames (ORFs) in lncRNAs and mRNAs. The median length of peptides translated by lncRNA ORFs was 51 aa, while for mRNA ORFs, it was 328 aa (Figures 1 and S1).

**Figure 2:**
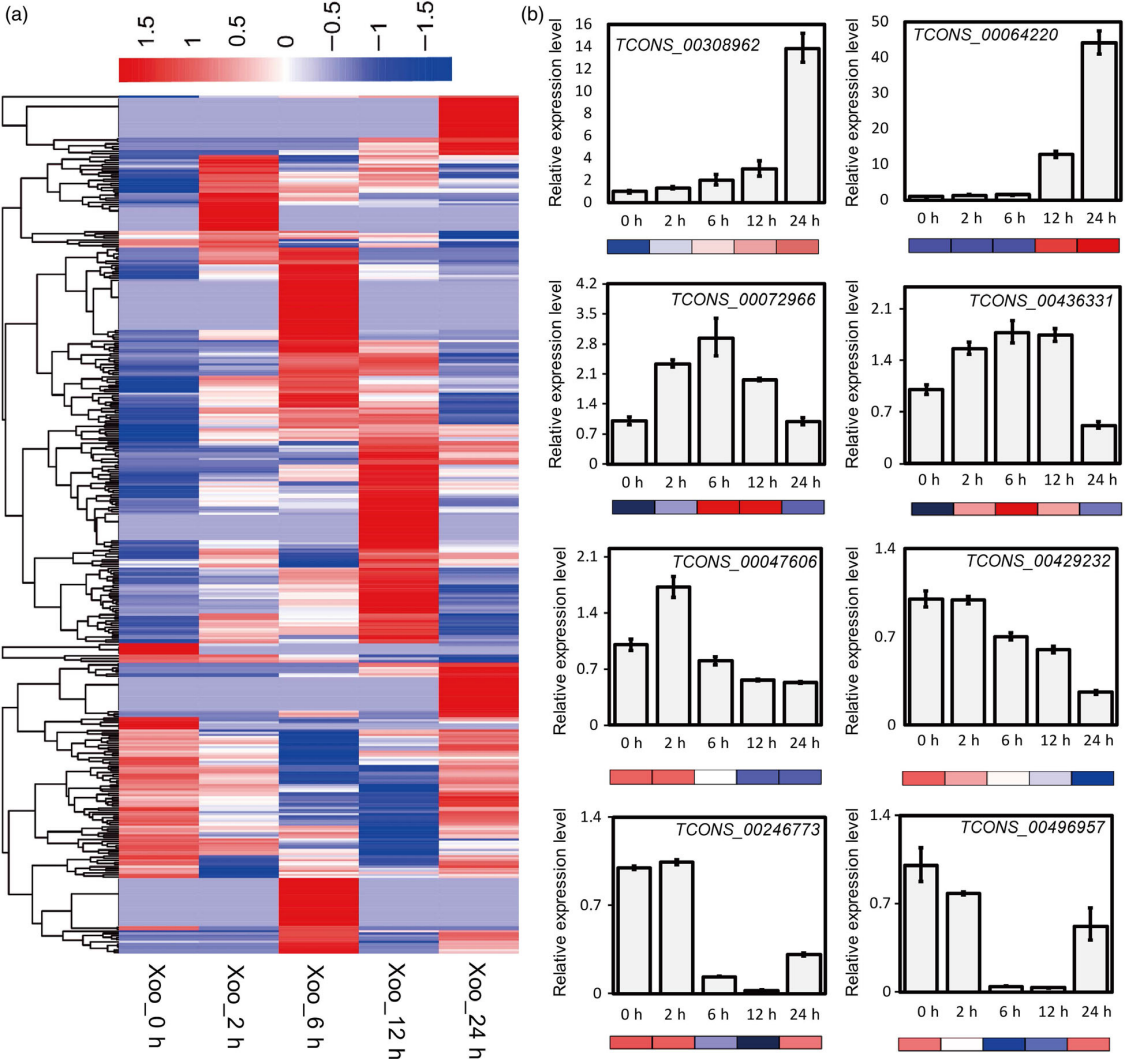
Expression profiles of rice lncRNAs responsive to *Xoo* infection. (a) Heatmap of differentially expressed lncRNAs in PXO99A‐inoculated rice leaves. All expression levels are normalized by FPKMs. *Xoo*_0h/*Xoo*_2h/*Xoo*_6h/*Xoo*_12h/*Xoo*_24h represent samples collected after *Xoo* treatment at 0, 2, 6, 12 and 24 hpi. *P* value < 0.05 and absolute value of fold change > 2 were set as the threshold for significantly differential expression analysis. (b) Validation of lncRNA expression profiles in RNA‐seq by quantitative real‐time PCR. Rice gene *Actin 2* was used as an endogenous control. Expression levels of *Xoo*_0h were set to 1. The data are expressed as the mean ± SD of three replicates.

To explore the transcriptional rewriting strategy of rice plants in the initiation of bacterial blight infection, we investigated the expression profile of lncRNAs based on the strand‐specific RNA sequencing data sets from rice flag leaves incubated with *Xoo* pathogens over time. The FPKMs of all lncRNAs detected in PXO99A‐infected leaves were analysed. The results showed that 567 lncRNAs expressed differentially in the five samples (Figure 2a). We scanned the distribution of these 567 lncRNAs in the 12 rice chromosomes and found that they are widely distributed in the rice genome, with no obvious location bias (Figure S2). The sequences and expression levels of responsive lncRNAs are listed in Table S1.

To confirm the reliability of deep sequencing data for lncRNA expression profiles, we detected the expression of eight lncRNAs with different expression patterns by qRT‐PCR. The results were consistent with those obtained from lncRNA sequencing, indicating that the sequencing data sets are reliable (Figure 2b). The above results reveal that these lncRNA transcripts may participate in the regulation of rice bacterial blight disease development or plant innate immunity.

### Targets analysis reveals *Xoo*‐responsive rice lncRNAs act as potential regulators of jasmonate pathway

**Figure 3:**
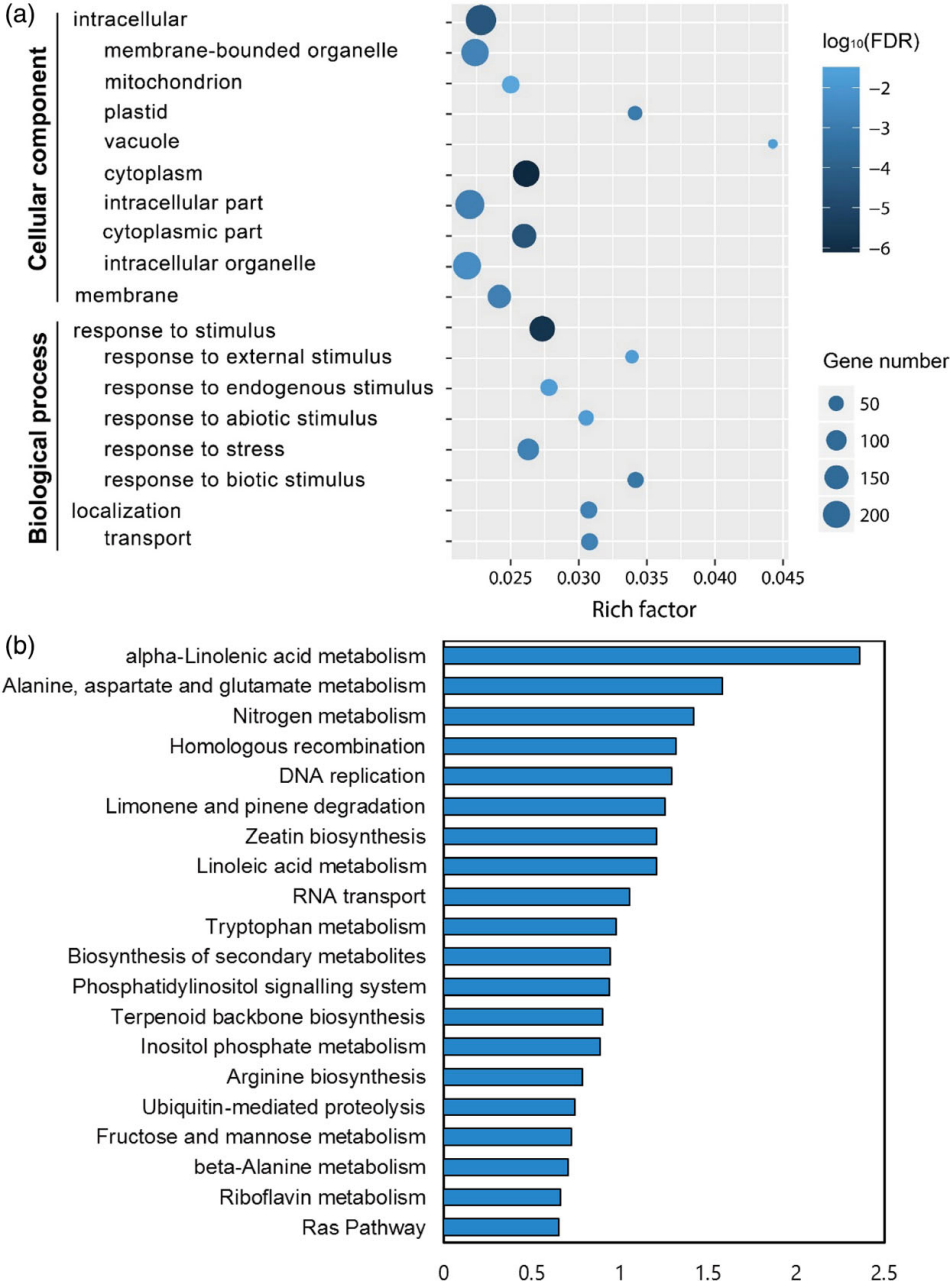
GO enrichment and KEGG pathway analyses of *cis* targets of *Xoo*‐responsive lncRNAs. (a) GO terms of 512 annotated genes in the GO database were analysed, and ‘cellular component’ and ‘biological process’ ontologies were enriched. Richness factor was calculated by the number of genes mapped to the GO term, and the log_10_(FDR) value indicates the significance of the GO term. (b) KEGG pathway enrichment analysis of 704 genes. The top 20 enriched pathways are shown. The *x*‐axis is –log_10_(*P* value), that is significance of KEGG pathway enrichment.

Epigenetic lncRNA regulation of gene expression includes *cis* and *trans* routes. *Trans*‐acting lncRNAs could influence gene expression in diverse biological processes at the transcriptional or post‐transcriptional level, while *cis*‐acting lncRNAs regulated genes epigenetically near themselves and only at the transcriptional level (Guil and Esteller, 2012). Decoding of *trans*‐acting mRNA targets of lncRNAs is based on lncRNA‐mRNA expression correlation analysis, which may generate massive amounts of promiscuous data (de la Fuente *et al*., 2004). To detect a reliable regulatory profile of *Xoo*‐responsive lncRNAs to rice protein‐coding genes, we combined the information of genomic location together with expression correlation analysis of lncRNAs and mRNAs. We firstly surveyed 100 kb upstream and downstream of the 567 differentially expressed lncRNAs for *cis* effect analysis and then performed lncRNA‐mRNA expression correlation analysis on the five data sets to narrow the *cis* regulation region. Seven hundred and forty‐four lncRNA‐mRNA matches comprising 282 lncRNAs and 704 mRNAs are remained, suggesting that some of these lncRNAs form *cis* regulatory pairs with multiple neighbouring genes (Table S2). A total of 512 of these 704 genes are annotated in the GO database. GO categories and subcategories were analysed, and 18 GO terms were found to be enriched in ‘cellular component’ and ‘biological process’ ontologies (Figure 3a). The result shows that most of these genes tend to function in the membrane and intracellular parts and are enriched in ‘response to stimulus’ (GO:0050896), which comprises five subcategories, including (i) ‘response to external stimulus’ (GO:0009605), (ii) ‘response to endogenous stimulus’ (GO:0009719), (iii) ‘response to abiotic stimulus’ (GO:0009628), (iv) ‘response to stress’ (GO:0006950) and (v) ‘response to biotic stimulus’ (GO:0009607), indicating that the genes in these terms are not only correlated with lncRNAs, but may also be associated with signal perception or transduction in plant innate immunity.

We next conducted a KEGG pathway enrichment analysis with these 704 genes, and the top 20 enriched pathways are shown in Figure 3b. The three most significant enriched KEGG pathways are (i) ‘α‐linolenic acid metabolism’ (KEGG entry: osa00592), (ii) ‘alanine, aspartate and glutamate metabolism’ (KEGG entry: osa00250) and (iii) ‘nitrogen metabolism’ (KEGG entry: osa00910). α‐Linolenic acid is a substance required for the biosynthesis of JA and methyl‐jasmonate (MeJA). The above *cis*‐acting analyses of lncRNAs have shown that the JA pathway were significantly enriched, strongly suggesting that lncRNAs may be new players in the modulation of JA pathway.

**Figure 4:**
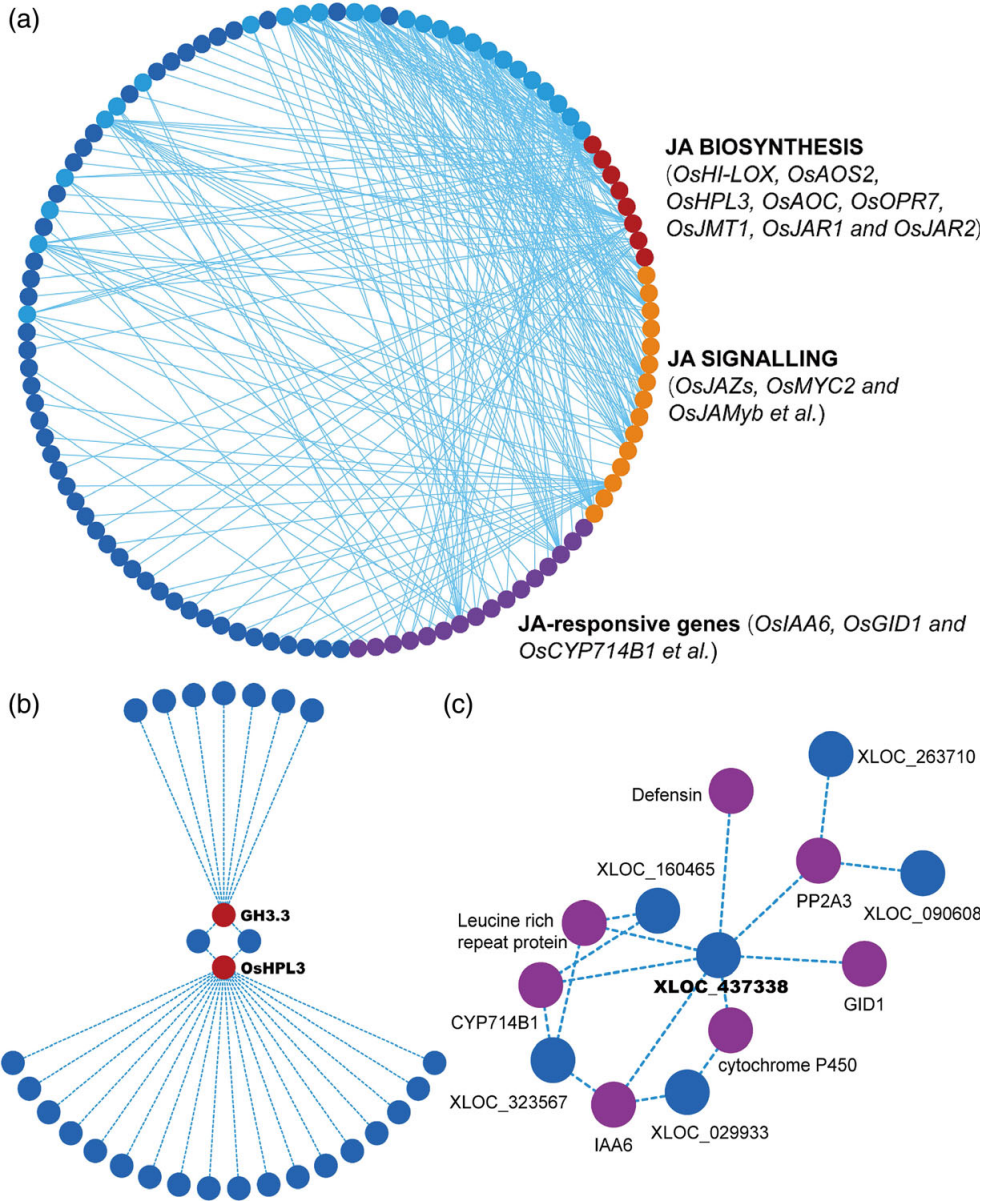
Regulatory network between lncRNAs and JA‐related mRNAs. (a) A full view of the interaction network between lncRNAs and JA‐related mRNAs. Circles in red, orange and purple represent rice genes previously reported to be related to the JA pathway, while blue circles are lncRNAs coexpressed with JA‐related mRNAs. The light blue circles highlight 26 lncRNAs significantly and complicatedly interconnected with the JA‐related genes. (b) An OsHPL3/GH3.3‐centred view of the interplays between lncRNAs. (c) An XLOC_437338‐centred view of the interplay between JA‐related mRNAs.

To further reveal the interplays between coding and noncoding regulators of JA pathway, we studied the coexpression of lncRNAs and JA‐related genes. We obtained mRNA expression profiles during *Xoo* incubation and found a variety of JA‐related genes differentially expressed, including the genes in JA biosynthesis, signalling and JA‐mediated output. To test whether these genes are associated with lncRNAs, we integrated lncRNA and mRNA expression data and analysed their correlation profiles. All lncRNA‐mRNA matches with a correlation coefficient more than 0.95 or less than −0.95 were remained. Finally, we screened 39 JA‐related genes as the potential targets of 73 lncRNAs (Table S3). The regulatory network was then constructed (Figure 4a). Among the 73 lncRNAs, 26 of them are significantly and complicatedly interconnected with JA‐related genes (Figure 4a, light blue circles). These 26 lncRNAs may be important in modulating JA pathways and are the candidates for further screening disease‐resistant mutants for functional studies. Detailed chromosome location and sequence information of these 26 lncRNAs are listed in Table S4.

Specifically, we found dozens of lncRNAs could simultaneously point to the same JA‐related gene*OsHPL3* (hydroperoxide lyase 3) (Figure 4b). *OsHPL3*, also known as *CYP74B2*, is reported to affect the levels of JA, green leaf volatiles and other volatiles and subsequently modulates rice‐specific defence responses against bacterial blight (Liu *et al*., 2012b; Tong *et al*., 2012). In addition, among the above‐mentioned 26 lncRNAs, we found XLOC_437338, which could interplay with 7 JA‐related genes and their downstream lncRNAs, implying a key regulatory role of this lncRNA in rice defence responses (Figure 4c). These observations suggest that a network constructed based on lncRNA‐associated JA‐related genes can provide an effective evidence for estimating gene functions, and lncRNAs may also participate in triggering JA‐mediated plant defence response.

### Phenotypic screening identified an lncRNA conferring resistance to rice bacterial blight

**Figure 5:**
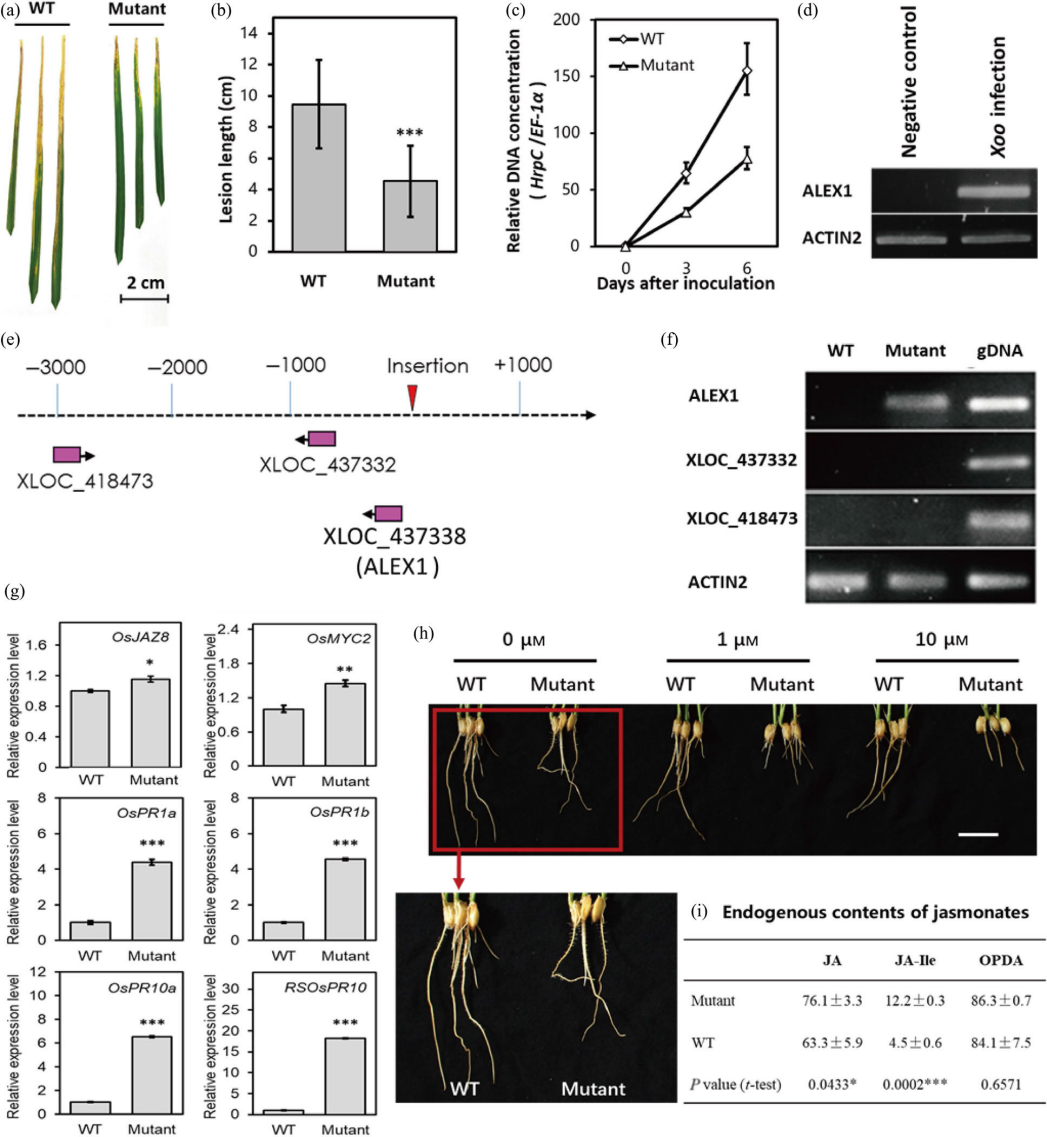
Phenotypic and molecular basis analysis of ALEX1 mutant. (a) Comparison of resistance to *Xoo* between wild type and ALEX1 mutant. (b) Lesion length of wild type and ALEX1 mutant after 14 days infected by PXO99A (*n* = 30). Asterisks indicate statistically significant differences compared with wild type by Student's *t* test (***P < 0.001). (c) Real‐time PCR analysis of relative DNA concentration between bacterial *HrpC* and rice *EF‐1*α in wild type and ALEX1 mutant. The data are expressed as the mean ± SD of three replicates. (d) The *Xoo‐*induced ALEX1 expression in rice leaves. (e) Model of enhancer trap insertion site and genes in the region 10 kb upstream or downstream of insertion site. (f) Expression levels of lncRNAs near the insertion site. Rice gene *Actin 2* was used as an endogenous control. The rice genomic DNA was also amplified as positive controls. (g) Relative expression levels of output genes in JA signalling. *Actin 2* was used as an endogenous control in quantitative real‐time PCR. The data are expressed as the mean ± SD of three replicates. Asterisks indicate statistically significant differences compared with wild type by Student's *t* test (*P < 0.05; **P < 0.01; ***P < 0.001). (h) Root development of ALEX1 mutant and WT plants under the treatment of a gradient of MeJA concentration. Scale bar = 2 cm. (i) Content of endogenous jasmonates in ALEX1 mutant and WT plants. The data are expressed as the mean ± SD of three replicates. Asterisks indicate statistically significant differences compared with wild type by Student's *t* test (*P < 0.05; ***P < 0.001).

To investigate whether the abnormal expression of these lncRNAs would influence rice disease defence, we applied the above identified 26 pathogen‐responsive and JA‐associated lncRNAs as candidates for screening disease‐resistant mutants in rice. We retrieved the worldwide main rice mutant database and identified four rice mutant lines with insertional mutations in two of the 26*Xoo*‐responsive lncRNAs: XLOC_285262 and XLOC_437338. We then treated the flag leaves of these mutants and their wild‐type rice plants with PXO99A for 14 days. However, only the mutant of XLOC_437338 exhibited rice blight resistance, while the mutants of XLOC_285262 showed no significant difference in blight lesion lengths (Figures 5a, b and S3). The relative expression of a conserved *Xoo* gene *hrpC* vs. the rice housekeeping gene *EF‐1*α also showed great suppression of *Xoo* growth in the XLOC_437338 mutant compared within the wild‐type plants (Figure 5c).

XLOC_437338 is specifically expressed in *Xoo*‐infected leaves (Figure 5d); therefore, we named this lncRNA as ALEX1 (*An Leaf Expressed and Xoo‐induced lncRNA 1*) in the following study. The ALEX1 mutant was constructed using an enhancer trap system, and the insertion site was 72 bp upstream of the sequencing‐annotated 5′‐end of ALEX1, which was positioned at the promoter region and was thought to have enhanced expression of this lncRNA (Figure 5e and S4). To confirm that disease resistance is an effect of the abnormal expression of ALEX1 but not any other noncoding genes, we scanned genes near the insertion site in detail. As shown in Figure 5e, we found two other lncRNAs located in the upstream of the insertion site. We then investigated the expression levels of these two lncRNAs and ALEX1 by RT‐PCR. The results showed that ALEX1 was not detectable in the wild‐type plants (WT), but dramatically up‐regulated in the mutant (Figure 5f). However, both XLOC_418473 and XLOC_437332 showed no expression in both WT and the mutant plants (Figure 5f). These observations indicated that the enhancer trap‐based T‐DNA insertion mutant indeed enhanced expression of ALEX1, and the resistant phenotype to bacterial blight is resulted from accumulated expression of ALEX1 rather than any other lncRNAs near the insertion site.

### JA pathway is activated in the ALEX1 mutant

ALEX1 is one of the 26 pathogen‐responsive and JA‐associated lncRNAs identified in the regulatory network (Figure 4c). Jasmonates are regarded as the main phytohormonal determinants of rice‐*Xoo* interaction (Dar *et al*., 2015). To explore whether JA signalling is altered in ALEX1 mutant, we first investigated JA‐responsive gene expression. The results showed JA‐related genes such as *JAZ8*,* MYC2, PR1a, PR1b, PR10a* and *RSOsPR10* were significantly up‐regulated (Figure 5g), indicating ALEX1‐mediated JA regulation in the modulation of rice disease responses.

Accumulating data suggest that JA could repress plant root growth (Merkouropoulos *et al*., 1999; Yang *et al*., 2013). We subsequently detected the root development status of the mutant and WT plants under the treatment of gradient concentration of MeJA. The results showed that the roots of ALEX1 mutant were shorter in length after 3 days treatment (Figure 5h), suggesting enhanced JA signalling in the ALEX1 mutant. The gradient concentration of the MeJA treatment assay also suggested that the ALEX1 mutant is more sensitive to MeJA than the wild type. These results show that JA signalling is activated with elevated ALEX1 expression in the rice mutant.

To correlate the activated JA signalling with endogenous levels of jasmonates, we performed LC‐MS/MS to determine the content of JA, JA‐Ile and OPDA in the ALEX1 mutant and WT plants (Figure 5i). The JA content increased in the ALEX1 mutant compared with that in the WT plants. In addition, the amino acid conjugate JA‐isoleucine (JA‐Ile), which is a major bioactive derivative of JA, accumulated up to threefold in mutant plants. The level of the JA precursor OPDA in ALEX1 mutant was slightly higher than that in WT, although with no statistical significance. These data suggest that the overall endogenous jasmonate content is up‐regulated in ALEX1 mutant, contextualizing the elevated JA signalling and enhanced resistance to bacterial blight.

### Overexpressing full length of ALEX1 in rice further confirmed activated JA pathway and resistance

**Figure 6:**
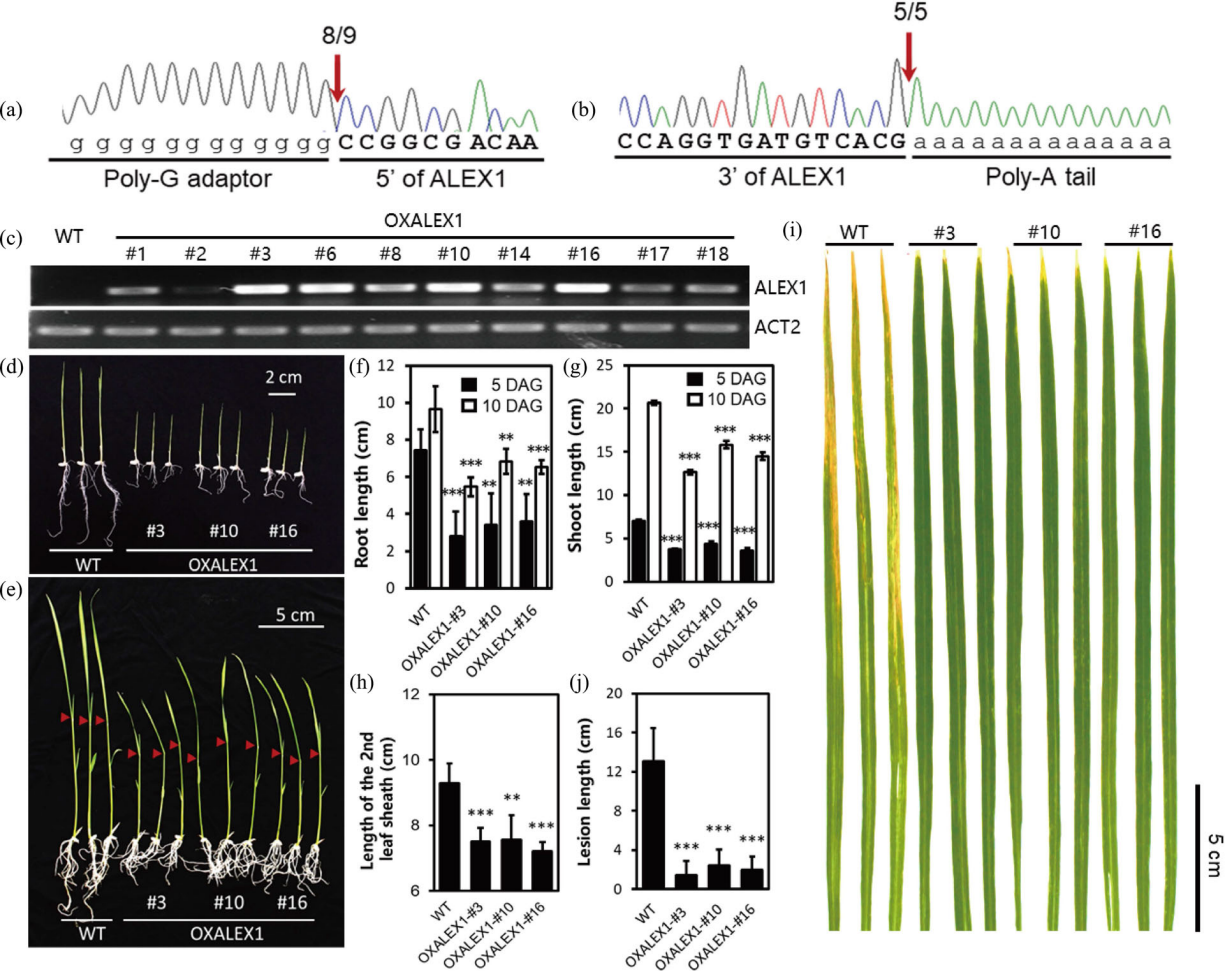
Full‐length characterization of ALEX1 and phenotype of ALEX1‐overexpression lines. (a and b) The full length of ALEX1 was investigated by 5′‐RACE (a) and 3′‐RACE (b) assays. (c) The expression level of ALEX1 in 10 OXALEX1 lines. The polymerase chain reaction is stopped after 32 cycles for ALEX1 and 26 cycles for *ACTIN2*. (d‐h) Seedlings of OXALEX1 and WT were photographed at 5 DAG (d) and 10 DAG (e), the length of root (f), shoot (g) and the second leaf sheath (h) were measured. Asterisks indicate statistically significant differences compared with wild type by Student's *t* test (**P < 0.01; ***P < 0.001; *n* = 15). (i) Comparison of resistance to *Xoo* between wild type and ALEX1‐overexpressing lines. (j) Lesion length of wild type and ALEX1‐overexpressing lines after 14 days infected by PXO99A (*n* = 15). Asterisks indicate statistically significant differences compared with wild type by Student's *t* test (***P < 0.001).

To further uncover the function of ALEX1, we performed its genomic loci analysis. Sequencing data showed *ALEX1* is located on the antisense DNA strand of rice Chromosome 8. We next performed rapid amplification of cDNA ends (RACE) to experimentally validate its 5′ and 3′ ends. The results evidenced that *ALEX1* locates at 22381757 bp to 22382050 bp of Chromosome 8 and transcribes an lncRNA of 294 nt in length (Figures 6a, b and S4). Based on the full‐length sequence, we overexpressed ALEX1 in rice and obtained 22 transgenic lines (OXALEX1). The expression level of ALEX1 in ten of these lines was detected by RT‐PCR (Figure 6c), among which, three lines with relatively higher ALEX1 expression levels were used for the following phenotype observation and resistance investigation. As shown in Figures 6d‐h, the root length, shoot length and the second leaf sheath length are all decreased in OXALEX1 plants when compared with WT, consistent with the enhanced effects of JA signalling. The plant inoculation with *Xoo* further evidenced increased disease resistance in OXALEX1 plants (Figures 6i and j). We also performed the infection assay by using another bacterial pathogen, *Xanthomonas oryzae* pv*. oryzicola* (*Xoc*), which causes bacterial leaf streak in rice. The result showed ALEX1 overexpressors can also be resistant to *Xoc* (Figure S5), suggesting that ALEX1 conferred broad‐spectrum resistance to rice bacterial pathogens.

**Figure 7:**
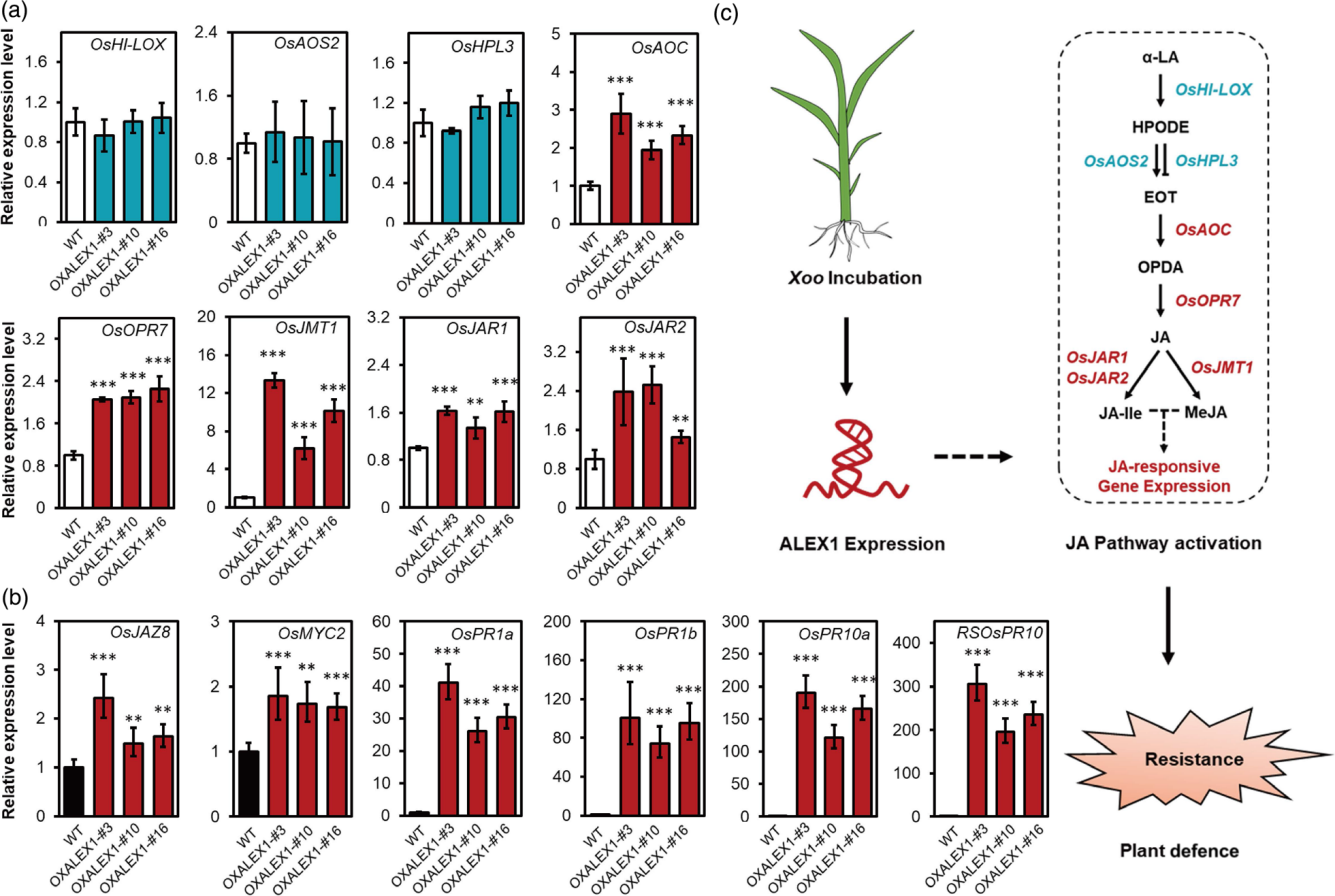
The expression of ALEX1 activates JA pathway and enhances resistance in rice. (a and b) Relative expression levels of genes function in JA biosynthesis (a) and signalling (b) of WT and ALEX1‐expressing lines. *Actin 2* was used as an endogenous control in quantitative real‐time PCR. The data are expressed as the mean ± SD of three replicates. Asterisks indicate statistically significant differences compared with wild type by Student's *t* test (**P < 0.01; ***P < 0.001). (c) A working model of the pathogen‐induced ALEX1 expression in activating JA pathway and rice defence.

To verify whether OXALEX1 plants could also activate the JA pathway, we then detected the expression of genes functioning in JA biosynthesis and signalling (Figure 7a and b). We found a couple of these genes are up‐regulated in OXALEX1, suggesting that both JA biosynthesis and signalling are activated by constitutive expression ALEX1.

The above observations evidenced the essential role of ALEX1 expression in regulating rice resistance to bacterial pathogens. To evaluate whether ALEX1 expression varied among different cultivars, we analysed natural variations of ALEX1 promoters in 3010 rice accessions described in the 3K Rice Genome Project (Wang *et al*., 2018a). Totally, 23 SNPs and two InDels were found in the 2000 bp upstream of ALEX1 (Table S5). We then performed phylogenetic and motif analysis by using the promoter sequences of 88 of 3010 rice accessions, which covered all of the above SNPs and InDels. The variations in addition or deletion of *cis* elements such as MYB binding or recognition site, MYC recognition site and dehydration‐responsive element (Figure S6) suggested that the transcription of ALEX1 may be varied in different rice accessions.

Taken together, we connected lncRNAs with the JA pathway in the regulation of plant innate immunity and experimentally confirmed the expression of an lncRNA ALEX1 could activate JA pathway and enhance rice resistance to bacterial blight (Figure 7c). Although the mechanism of how ALEX1 affects JA pathway is yet to be determined, we highlighted the biological function of ALEX1 in JA pathway and unveiled its role in rice defence. The noncoding RNA resources identified in this study may have a significant potential for plant disease research and crop breeding.

## Discussion

Plant lncRNAs have been identified in Arabidopsis (Liu *et al*., 2012a), rice (Zhang *et al*., 2014), maize (Li *et al*., 2014), wheat (Xin *et al*., 2011), cotton (Wang *et al*., 2015a; Zhang *et al*., 2018) and Medicago (Wang *et al*., 2015b). Most of these studies demonstrate that lncRNAs play roles in plant vegetative growth and reproductive development, while limited data are available for plant lncRNA regulation of disease resistance and biotic stress response. In this study, we performed strand‐specific paired‐end deep sequencing of five rice leaf samples collected after *Xoo* treatment at different time points and obtained 48,727 high‐confidence putative lncRNAs. lncRNAs in plants were revealed to be relatively shorter in length and lower in expression than protein‐coding mRNAs (Chekanova, 2015). Our data showed that approximately 98% of lncRNAs we identified in rice leaves were 200–500 nt in length, far below that of mRNAs. Most lncRNAs are single‐exon transcripts, and the FPKM distribution also showed their relatively lower expression compared with mRNAs. These results are consistent with previously reported lncRNA characteristics in rice and other organisms.

We screened 567 lncRNAs that responded to infection of *Xoo*, most of which were expressed in chronological order (Figure 2), indicating that the transcriptome rearrangement of rice leaves experienced gradual change during the invasion and reproduction of bacterial pathogens. Several lncRNAs showed sustained upward or downward expression trends, suggesting they might be bacteria dosage or time course‐dependent regulators. The others, which were first up‐regulated or down‐regulated at a certain time point and finally regressed to a similar level at 12 or 24 hpi to the zero point, may exert their function intermediately in the signal transduction of stress.

Phytohormones, such as JA and SA, have been shown to be involved in plant–pathogen interactions. The signalling pathways of phytohormones in rice innate immunity have mostly been studied using interaction models of rice with the bacterial pathogen *Xoo* and fungal *M*. *oryzae* systems. Analysis of the GO and KEGG pathways of lncRNA‐targeted genes could give a peripheral description of the lncRNA function we focused on. The most significantly enriched pathway, alpha‐linolenic acid metabolism, indicates that lncRNAs may associate with the accumulation of jasmonates after *Xoo* infection. Nitrogen and amino acid metabolism are thought to alter JA biosynthesis process, while emerging reports also suggest that JA could also induce rapid changes in carbon and nitrogen dynamics (Appel *et al*., 2012; Gomez *et al*., 2010; Schmelz *et al*., 2003). The GO and KEGG pathway analyses of *cis*‐acting mRNAs in *Xoo*‐responsive rice lncRNAs distinctly demonstrated that JA, the vital defence‐responsible phytohormone, contributes to lncRNA‐mediated defence response or plant innate immunity.

Recent progresses have shed light on the emerging role of plant lncRNAs as important players for regulating phytohormones. For instance, ELENA1 enhances plant resistance against *Pseudomonas syringe* pv. *tomato* DC3000 by directly interact with the transcriptional coactivator MED19a and affects enrichment of MED19a on the *PR1* promoter (Seo *et al*., 2017). The activated *PR1* expression may also crosstalk with phytohormone signals. Silencing of two cotton lncRNAs conferred enhanced resistance towards *V. dahliae* and *Botrytis cinerea*, which is thought to be attributed to the increased expression of *LOX1* and *LOX2*, suggesting the JA biosynthesis would be affected by lncRNA expression in cotton (Zhang *et al*., 2018). In this study, we identified an lncRNA, ALEX1, which can be specially induced after pathogen infection. Expression of this lncRNA in rice exerts activated JA signalling and increased endogenous JA levels, conferring broad‐spectrum resistance to the bacterial pathogens. We also performed natural variation and phylogenetic analysis of ALEX1 promoters in 3010 rice accessions. Motif analysis revealed addition or deletion of *cis* elements in the ALEX1 promoters of different cultivars, indicating the potential to identify disease resistance cultivars with high expression level of ALEX1.

Previous studies have well‐characterized lncRNAs in plants, while research on the function of plant lncRNAs is still in its infancy due to relatively low expression levels, diverse and complex functional mechanisms and less conserved sequences among different species (Chekanova, 2015). In this study, we showed that both the biosynthesis and signalling of JA pathway are activated by elevated ALEX1 expression in rice, indicating that ALEX1 may be an upstream regulator of this biological process. Although we described the general regulation between ALEX1 and JA pathway, the molecular mechanism behind this is still unclear. These concerns give rise to possible clues exploring the mechanism of the ALEX1‐modulated downstream pathway. Further studies and research efforts are ongoing to discover the epigenetic regulation of this lncRNA at the molecular level.

## Experimental procedures

### Plant materials and growth conditions

The rice cultivar Zhonghua 11 (*Oryza sativa* L. ssp. *japonica*) was used in this study. Rice seeds were imbibed in darkness for 2 day at 32 °C and then grown for approximately 2 weeks in a soil seedbed at 27 °C and 70% humidity with a photoperiod of 10‐h light and 14‐h dark. The seedlings were then transplanted to a field, and plants were maintained under routine management practices during the rice‐growing season. The rice insertional mutants of lncRNAs were collected from both the Rice Mutant Database (Zhang *et al*., 2006) and POSTECH (Jeon *et al*., 2000). The 294 nt full‐length sequence of ALEX1 was amplified and subcloned into the binary vector pRHV (He *et al*., 2018). The resulting plasmid of pRHV‐ALEX1 was then transformed into Zhonghua 11. Transgenic lines were selected in MS medium with 50 mg/L hygromycin B (Biofroxx, Germany). The T2 generation of OXALEX1 lines is used for the detection of gene expression and phenotype observation.

### Library construction and sequencing

Samples were collected at 0, 2, 6, 12 and 24 hpi. Leaf fragments approximately 2 cm in length that were immediately adjacent to the inoculation site were collected. Total RNA was obtained from the samples for sequencing.

The construction of transcriptome libraries and deep sequencing were performed by the Novogene Corporation (Beijing). Ribosomal RNA was removed using an Epicentre Ribo‐Zero^™^ Kit (Epicentre Technologies Corp., Chicago, IL). Subsequently, random hexamers were used as primers to amplify cDNA with the templates of fragmented RNAs. Libraries were quality‐controlled and quantified using the BioAnalyzer 2100 system and qPCR (Kapa Biosystems, Woburn, MA). The resulting libraries were sequenced on an Illumina HiSeq 2000 instrument that generated paired‐end reads of 100 nt.

### lncRNA identification

The clean transcript reads without adaptors were mapped independently to the rice genome using the TopHat 2.0 program (Trapnell *et al*., 2012). The transcriptomes from each data set were assembled using the CUFFLINKS package according to the instructions provided (Trapnell *et al*., 2010). According to the sequence and structural information of lncRNAs, we set a primary screening strategy that comprised the following five key steps: (i) the nearest distance of lncRNAs should be more than 500 bp, with other transcripts for single‐exon transcript; (ii) lncRNAs should be longer than 200 nt; (iii) the expression level for single‐exon lncRNAs should be FPKM ≥ 2, and for multi‐exon lncRNAs, FPKM ≥ 0.5; (iv) the known non‐lncRNAs, such as the precursor of microRNAs, pseudogenes, rRNAs, tRNAs, snRNAs and snoRNAs, should be filtered; and (v) classification of candidate lncRNAs into lincRNAs, intronic lncRNAs and antisense lncRNAs should be compared using cuff compare analysis. After these five procedures, the remaining transcripts were further filtered for a test of protein‐coding potential. All transcripts with CPC scores > 0 or for which pfam scan tool search yielded even one protein domain hit were discarded.

### Expression analysis

The expression analysis for RNA sequencing data was quantitated using cuff diff to calculate FPKMs (fragments per kilobase of exon per million fragments) for lncRNAs and mRNAs in each data set. To screen differentially expressed lncRNAs, a *P* value < 0.05 and the absolute value of fold change >2 were set as the threshold for significantly differential expression analysis.

The expression levels of lncRNAs and mRNAs were experimentally detected by quantitative or semi‐quantitative RT‐PCR. Real‐time PCR was performed using the SYBR Premix Ex Taq^™^ Kit (Takara) following the manufacturer's instructions. The rice *ACTIN2* gene was used as a reference gene to standardize RNA samples. All reactions were run in triplicate. Relative expression levels were calculated using the 2^−▵▵CT^ model, and the results are presented as the mean ± standard deviation. All primers used in this study are listed in Table S6.

### GO enrichment and KEGG pathway analysis

To classify the genes that were putative *cis* targets of lncRNAs, the Gene Ontology (GO) term was used to perform the classification analysis. The AgriGO (http://bioinfo.cau.edu.cn/agriGO/) program was used for this analysis, and the terms ‘biological process’, ‘molecular function’ and ‘cellular component’ were defined with default parameters. *Oryza sativa* 7.0 non‐TE was selected as the background for selecting references. The KEGG pathway of *cis*‐acted lncRNA target genes was analysed using KOBAS (http://kobas.cbi.pku.edu.cn/) software.

### Network construction

The JA biosynthesis‐ and signalling‐associated rice genes, with annotations, were retrieved from the literature, and we collected 39 genes to study their interactions with lncRNAs. The mRNAs with correlation coefficients above 0.95 or below −0.95 were considered the targets of lncRNAs. We then visualized the lncRNA‐mRNA matches as a network using Cytoscape (http://cytoscape.org/) software.

### The RACE analysis

The RNA samples of *Xoo*‐infected rice leaves are extracted and stored at −80 °C. The 5′ end of ALEX1 was amplified by using classic RACE described in reference (Scotto‐Lavino *et al*., 2006). Briefly, the purified cDNA products of reverse transcription were tailed using terminal deoxynucleotidyl transferase and dCTP (Takara, Dalian). The 13 nt poly(G) was used as the forward primer in the following nested PCR assays. The 3′ end of ALEX1 was amplified by using 3′‐Full RACE Core Set Kit (Takara, Dalian) following the manufacturer's instructions.

### Plant inoculations and pathogen growth

Rice flag leaves were inoculated with the *Xoo* strain PXO99A by the leaf‐clipping method. Briefly, the leaf tip is cut with scissors previously dipped in PXO99A bacterial suspension with an OD600 of 0.2. Approximately 20‐cm‐long infected leaf segments of the WT and XLOC_437338 mutant were collected 0, 3 and 6 days after infiltration. The DNA samples were extracted, and the gene copy numbers of bacterial *hrpC* and rice *EF‐1*α were quantitated using real‐time PCR, as described, with modifications (Ross and Somssich, 2016). The primer sequences for amplifying *HrpC* and *EF‐1*α are listed in Table S5. The blight lesion lengths were measured 14 days after infiltration. The blight lesion length data represent the mean ± SD of more than 10 rice plants. To detect the resistance of rice to bacterial leaf streak disease, Flag leaves were inoculated with *Xoc* strain XT8 by the penetration method using a syringe (Ke *et al*., 2014). Lesions were photographed and measured at 7 days after inoculation.

### MeJA treatment

The rice seeds of the wild‐type Zhonghua 11 and the ALEX1 mutant were germinated in water at 32 °C for 3 days. Methyl‐jasmonate (Sigma‐Aldrich #392707) was diluted in de‐ionized water to the designated concentrations and used for the treatment of the above 3‐day‐old seedlings. The root status was photographed after 6 days of cultivation in a climate chamber at 27 °C and 70% humidity.

### Determination of JA and derivatives

JA, JA‐Ile and OPDA were analysed by liquid chromatography coupled to mass spectrometry (LC‐MS/MS), as previously described (Chen *et al*., 2012). Briefly, the upmost leaf of 3‐week‐old rice plants was homogenized in liquid nitrogen. A 200‐mg sample of powdered leaves of fresh tissue was added to 800 μL extraction buffer comprising isopropanol: water: HCl at 1000 : 500 : 1, and the internal standard was 10 ng H_2_JA (Cerilliant). The mixtures were agitated for 30 min at 4 °C, followed by addition of 1 mL CH_2_Cl_2_, with 30‐min gentle agitation. After 10 min of centrifugation at 13 000 ***g***, at 4 °C, approximately 900 μL solvent from the lower phase was collected; 100 μL of 60% methanol was added to the sample, and 10 μL of each was injected onto a C18 column for further determination via LC‐MS/MS (AB Sciex Q‐TOF 5600 + system).

## Accession numbers

The strand‐specific whole transcriptome sequencing of Xoo‐infected rice samples generated in this study was submitted to NCBI Sequence Read Archive database with the BioProject ID PRJNA544880.

## Authors’ contributions

YY and YFZ carried out the functional analysis and drafted the manuscript. YZF, HH and JPL carried out mutant screening and validation experiments. YWY and MQL performed qRT‐PCR validation. YCZ analysed data. YQC conceived of the study, and participated in its design and coordination and helped to draft the manuscript. All authors read and approved the final manuscript.

## Competing interests statement

The authors declare that they have no competing financial interests.

## Supporting information


**Figure S1**. Sequence and structural comparison of rice lncRNAs and mRNAs identified in this study.
**Figure S2.** Chromosome distribution of *Xoo*‐responsive lncRNAs.
**Figure S3.** Comparison of the *Xoo* resistance of lncRNA insertional mutants and WT.
**Figure S4.** The genomic loci analyses of ALEX1 and its mutant.
**Figure S5.** Comparison of resistance to *Xoc* between wild‐type and ALEX1‐overexpressing lines.
**Figure S6.** Natural variation and phylogenetic analysis of ALEX1 promoter.

**Table S1.** Expression of 567 *Xoo*‐responsive lncRNAs.
**Table S2.** Expression profile of 704 *cis*‐acting mRNAs.
**Table S3.** Expression correlation of 73 lncRNAs and 39 JA‐related genes.
**Table S4.** Chromosome location and sequence information of 26 lncRNAs that are interconnected with JA‐related genes.
**Table S5.** Natural variation of ALEX1 and its promoter region in 3,010 rice accessions.
**Table S6.** Primers used in this study.
