# Supplementary material for: Transcriptional landscape of pathogen‐responsive lncRNAs in rice unveils the role of ALEX1 in jasmonate pathway and disease resistance
Source: Plant Biotechnol J. 2019 Sep 11;18(3):679–90. doi: 10.1111/pbi.13234 (PMC7004900; doi:10.1111/pbi.13234)
Supplement: Supplementary file 1 — Figure S1. Sequence and structural comparison of rice lncRNAs and mRNAs identified in this study. Figure S2. Chromosome distribution of Xoo‐responsive lncRNAs. Figure S3. Comparison of the Xoo resistance of lncRNA insertional mutants and WT. Figure S4. The genomic loci analyses of ALEX1 and its mutant. Figure S5. Comparison of resistance to Xoc between wild‐type and ALEX1‐overexpressing lines. Figure S6. Natural variation and phylogenetic analysis of ALEX1 promoter. [file PBI-18-679-s002.docx]

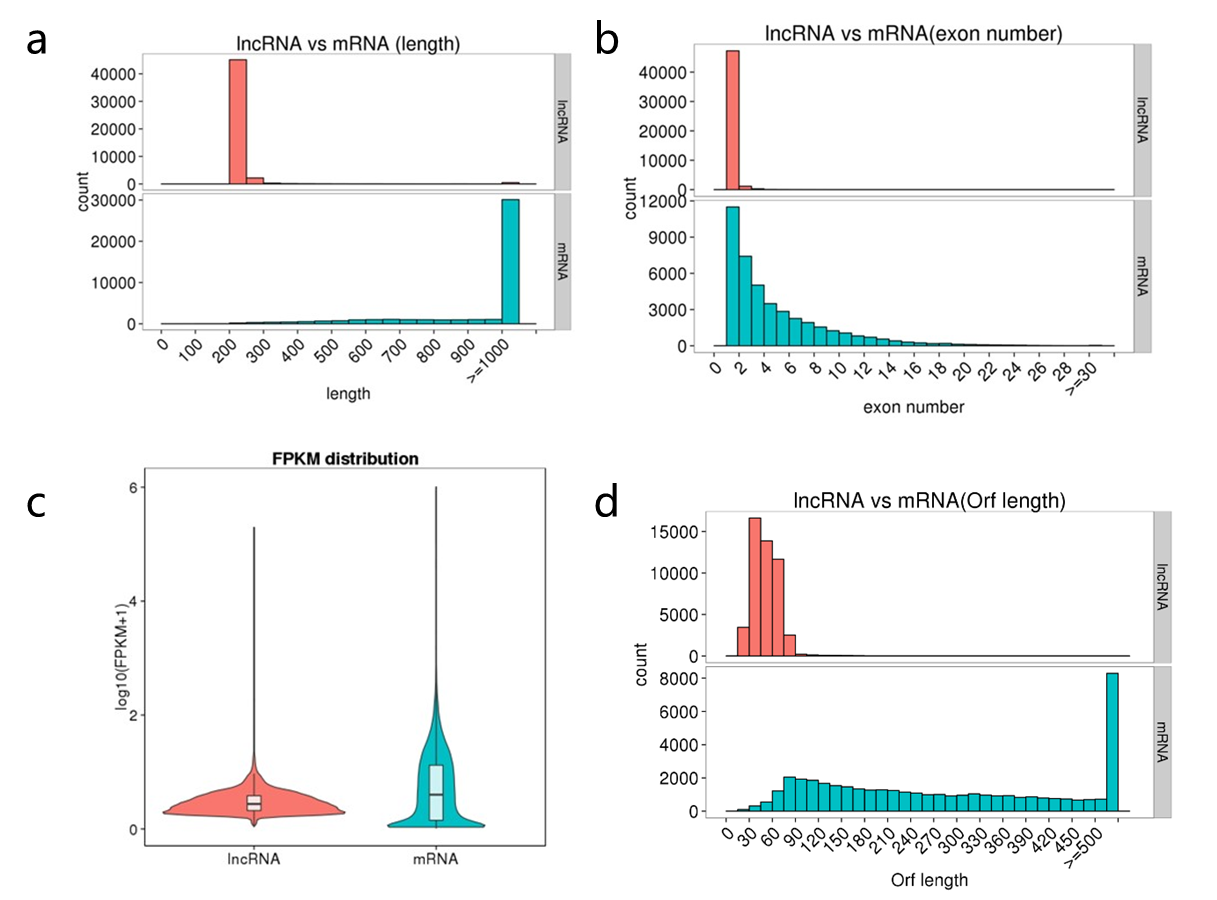
 **Figure S1***.* **Sequence and structural comparison of rice lncRNAs and mRNAs identified in this study.** The figure shows the different characteristics of lncRNAs vs. mRNAs identified in the *Xoo*-infected rice flag leaves. (a) RNA length, (b) exon numbers, (c) FPKM distribution and (d) length of ORFs.


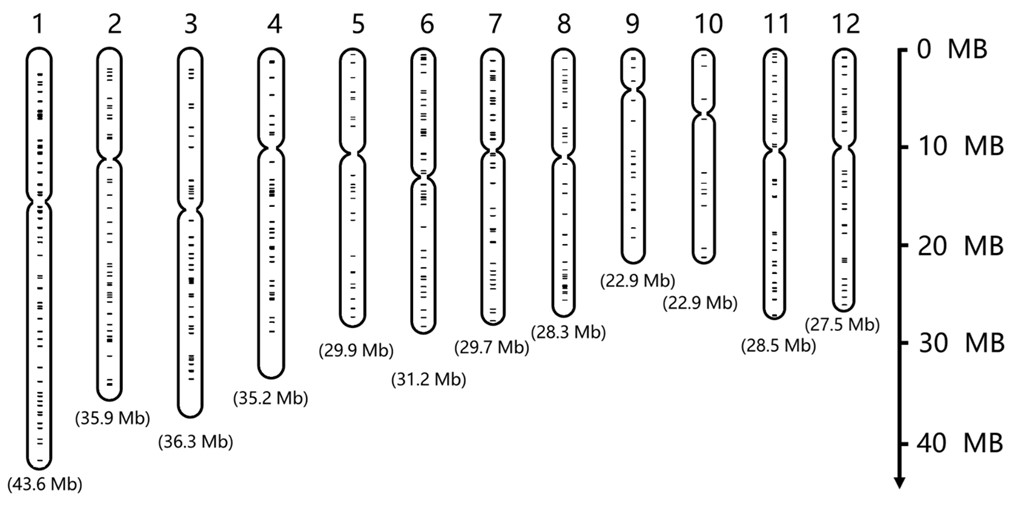
**Figure S2. Chromosome distribution of *Xoo*-responsive lncRNAs.** The DNA loci of 567 lncRNAs that have differentially expression in response to PXO99A infection were marked in the 12 rice chromosomes.


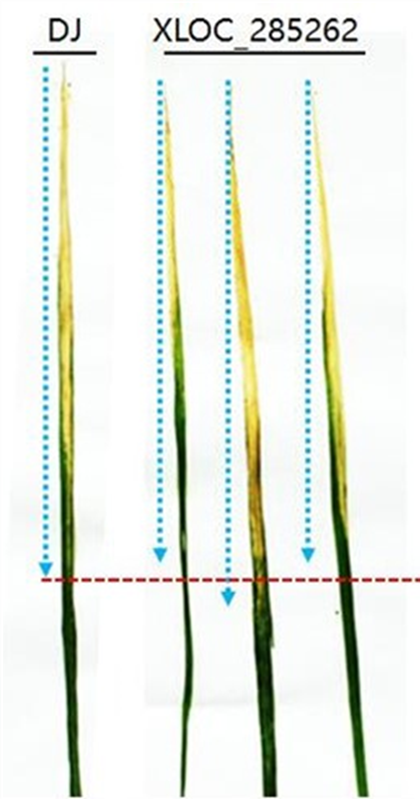


**Figure S3.** **Comparison of the *Xoo* resistance of lncRNA insertional mutants and WT.** 3 rice mutant lines with insertional mutations in XLOC_285262. Flag leaves of these mutants and their wild-type Dongjing (DJ) rice plants were infected with PXO99A for 14 days. The rice mutants were collected from POSTECH and the Mutant ID from left to right are 1B-11726L, 3A-17685, 3D-50428.


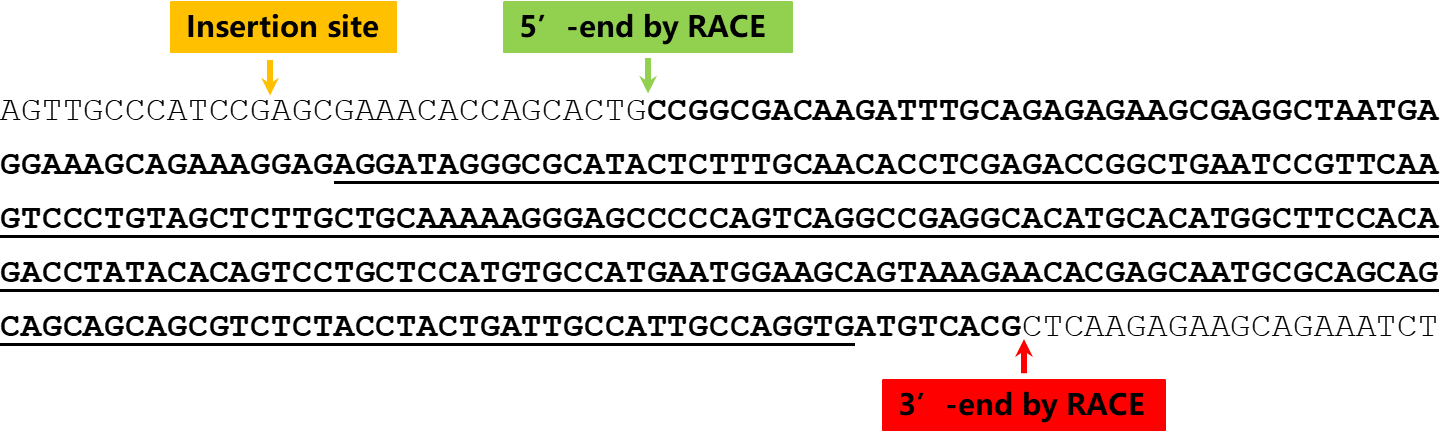


**Figure S4. The genomic loci analyses of *ALEX1* and its mutant.** The underlined is sequencing-annotated 209 nt ALEX1 transcript, while the one in bold represents the 294 nt full-length sequence of ALEX1 verified by RACE assays. The enhancer-trap based T-DNA insertion site of ALEX1 mutant located at the promoter region of *ALEX1*, which conferred elevated expression of this lncRNA in rice.


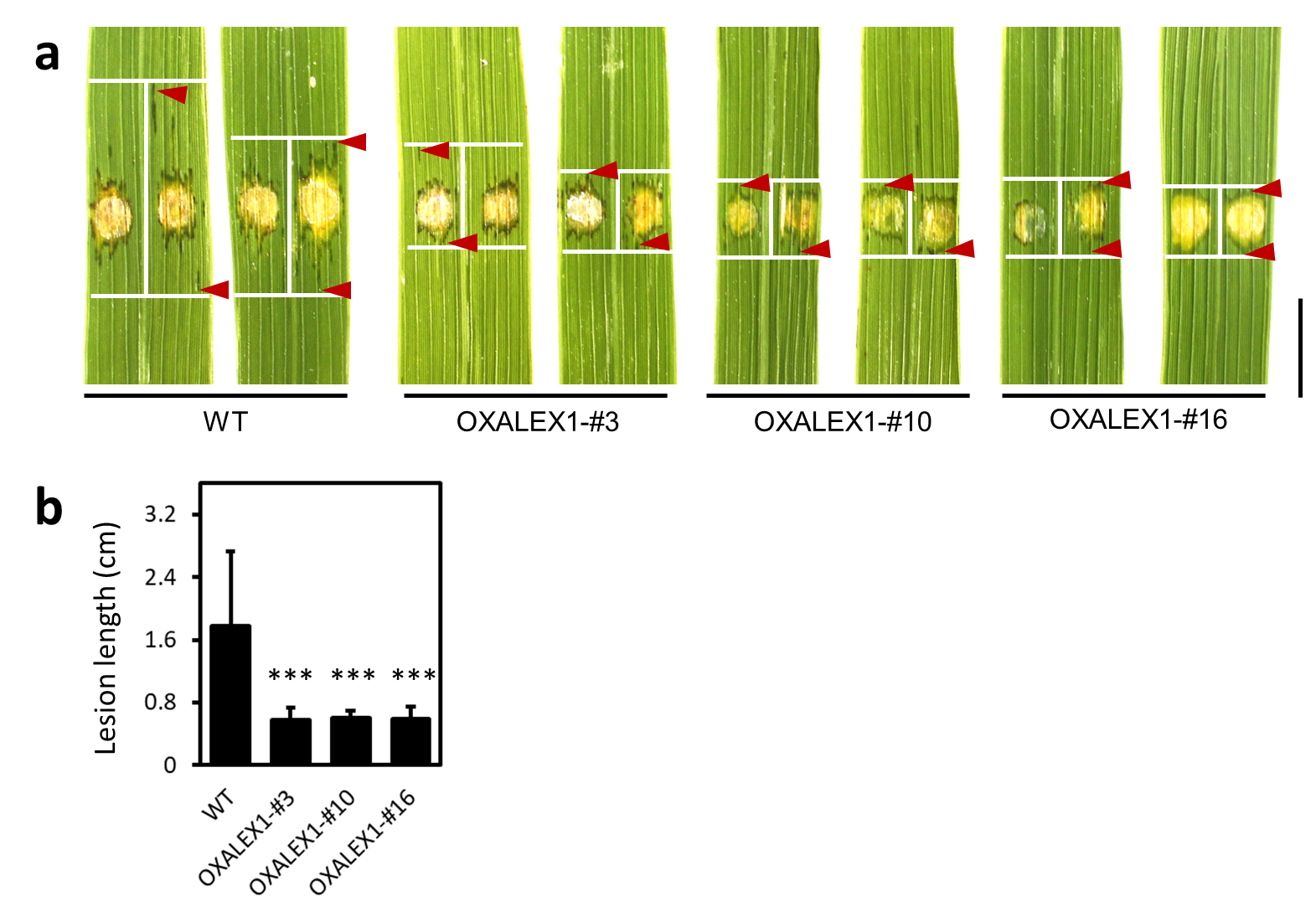


**Figure S5. Comparison of resistance to *Xoc* between wild-type and ALEX1-overexpressing lines.** Flag leaves of WT and OXALEX1 were infected with *Xanthomonas oryzae* pv. *oryzicola* strain XT8 and were photographed after 7 days inoculation. (a) Phenotype of *Xoc* infection, scale bar=1 cm. Triangles in red indicates lesions along the leaf veins. (b) Lesion length of wild-type and ALEX1-overexpressing lines after 7 days infected by *Xoc* (n=15). Asterisks indicate statistically significant differences compared with wild-type by Student's t-test (***P <0.001).

a


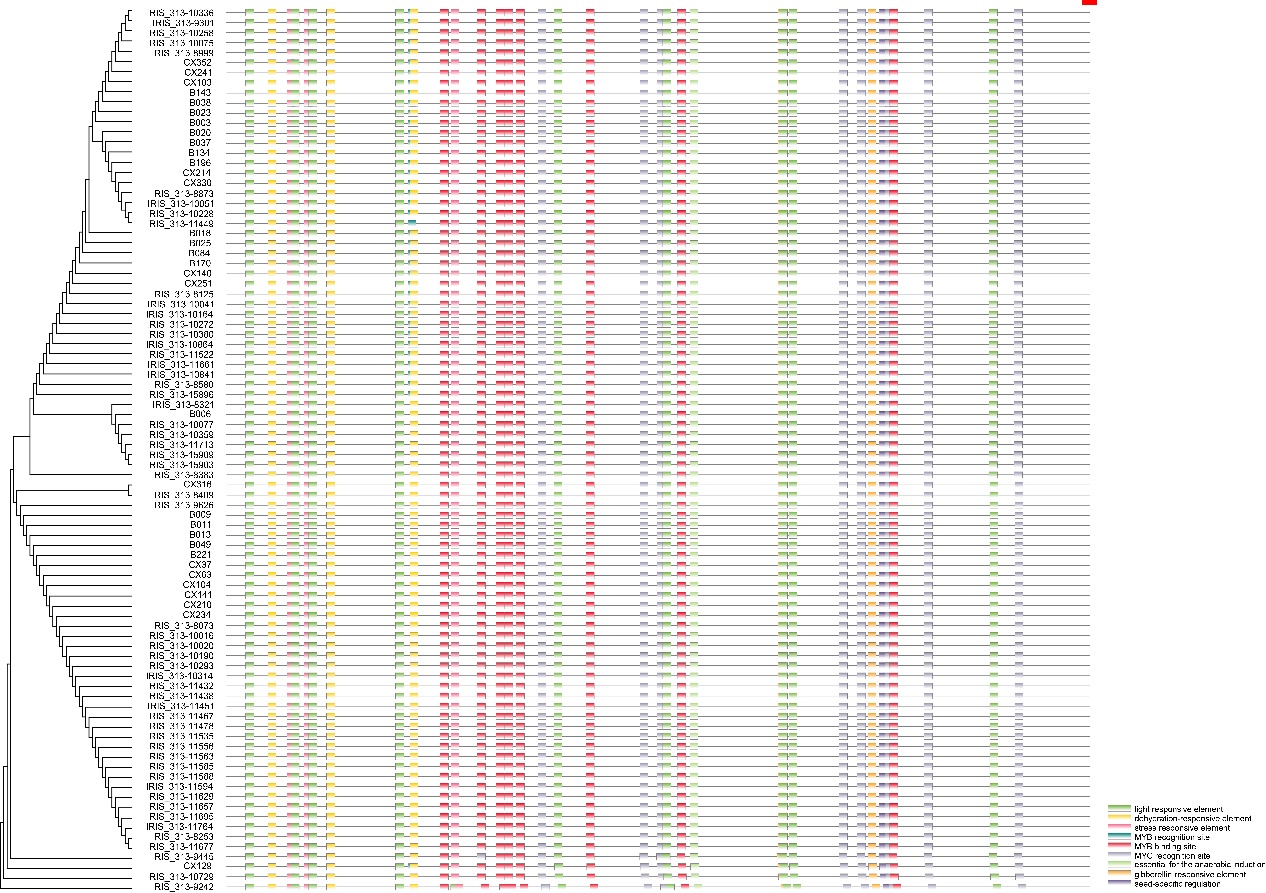


b


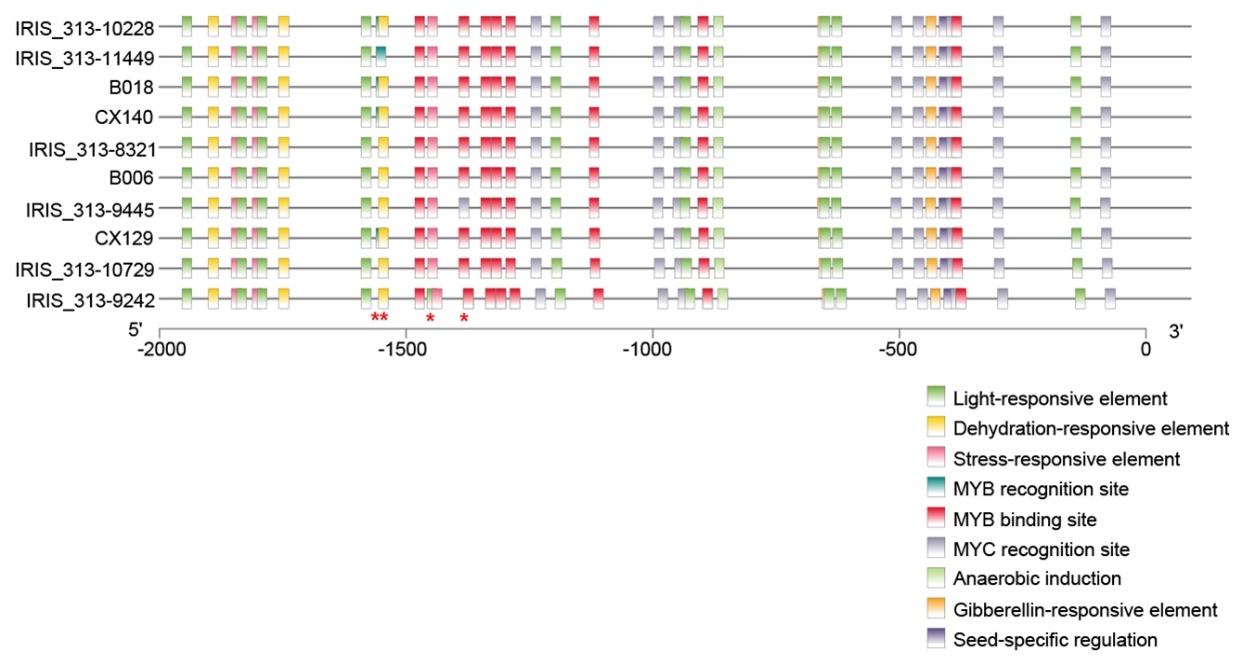


**Figure S6. Natural variation and phylogenic analysis of ALEX1 promoter.** Phylogenic analysis of ALEX1 promoter region based on 88 of 3,010 rice accessions described in the 3K Rice Genome Project. (a) Phylogenic and motif analysis of ALEX1 promoter. (b) Motif analysis of 10 rice cultivars showed differences in *cis* elements added or deleted in the upstream of ALEX1.
